# Exploring Copper’s role in stroke: progress and treatment approaches

**DOI:** 10.3389/fphar.2024.1409317

**Published:** 2024-09-26

**Authors:** Gang Peng, Yongpan Huang, Guangdi Xie, Jiayu Tang

**Affiliations:** ^1^ The School of Clinical Medicine, Hunan University of Chinese Medicine, Changsha, Hunan, China; ^2^ Department of Neurology, Brain Hospital of Hunan Province, Changsha, Hunan, China; ^3^ School of Medicine, Changsha Social Work College, Changsha, Hunan, China; ^4^ Department of Neurology, Huitong People’s Hospital, Huitong, Hunan, China

**Keywords:** copper ions, cerebral ischemia/reperfusion injury, cuproptosis, apoptosis, oxidative stress

## Abstract

Copper is an important mineral, and moderate copper is required to maintain physiological processes in nervous system including cerebral ischemia/reperfusion (I/R) injury. Over the past few decades, copper induced cell death, named cuprotosis, has attracted increasing attention. Several lines of evidence have confirmed cuprotosis exerts pivotal role in diverse of pathological processes, such as cancer, neurodegenerative diseases, and I/R injury. Therefore, an in-depth understanding of the interaction mechanism between copper-mediated cell death and I/R injury may reveal the significant alterations about cellular copper-mediated homeostasis in physiological and pathophysiological conditions, as well as therapeutic strategies deciphering copper-induced cell death in cerebral I/R injury.

## Introduction

Copper is an essential trace nutrient required for various cellular functions, meanwhile, copper accumulation beyond cellular needs is toxic (Khalfaoui-Hassani et al., 2021). Copper exists in two states, Cu^+^ and Cu^2+^, in body. In bodily fluids, copper is predominantly in the form of Cu^2+^, while inside cells, primarily exists as Cu^+^ (Kucková et al., 2015). Under the action of oxidoreductases, a conversion between Cu^+^ and Cu^2+^ existed, which electron transfer occurs through the Fenton reaction, leading to the generation of ROS, including superoxide anion (O^2−^), nitric oxide (NO-), hydroxyl radical (OH), and hydrogen peroxide (H_2_O_2_) (Valko et al., 2005). ROS could oxidize and damage biological molecules, including proteins, nucleic acids, and lipids. Additionally, ROS could interfer with the synthesis of iron-sulfur clusters (Slezak et al., 2021). Copper, assimilated from the digestive tract, undergoes hepatic metabolism to form ceruloplasmin, the primary copper-containing protein in the bloodstream, extensively present in diverse bodily organs. Ceruloplasmin is responsible for transporting 95% of copper in the bloodstream, making it a reliable marker for assessing the body’s copper levels (Piacenza et al., 2021). The diagnosis of specific copper metabolism disorders involves evaluating internal copper levels through the measurement of ceruloplasmin (CP) content (Bandmann et al., 2015). When ceruloplasmin (CP) amounts to the surface of target cells, which interacts with its corresponding receptors to release copper. The released copper is then absorbed and utilized by the targeted cells. The binding and release of copper ions by CP allow for distinct distribution of copper in multiple tissues and organs (Liu Z. et al., 2022). However, copper not bound to ceruloplasmin could still be continuously absorbed by tissues in the blood. Ceruloplasmin could oxidize ferrous ions (Fe^2+^) to ferric ions (Fe^3+^), facilitating the transportation of iron in the plasma, which plays a crucial role in maintaining the iron homeostasis in living organisms (Raia et al., 2023). During the oxidation of ferrous iron to ferric iron, ceruloplasmin facilitates the transfer of ferric iron into transferrin, preventing oxidative stress damage caused by the Fenton reaction (Vasilyev, 2019). Therefore, ceruloplasmin possesses certain antioxidant capabilities, manifested through its oxidase activity towards low-valent metal ions and glutathione peroxidase. It also exhibits the ability to eliminate reactive oxygen species (ROS) (Liu Z. et al., 2022; Samygina et al., 2017).

**FIGURE 1:**
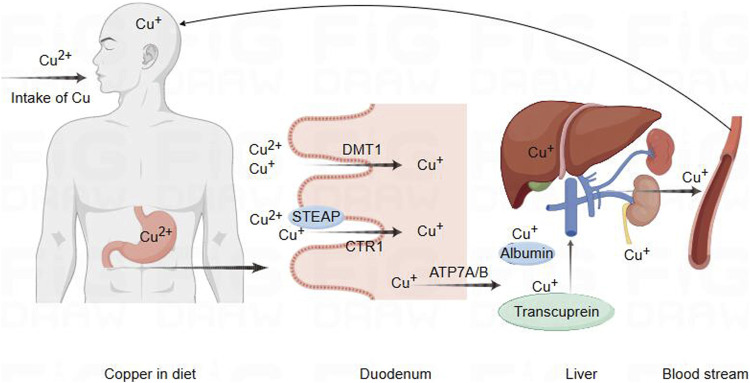
Schematic diagram of copper ion regulation in cells for cellular function. Copper metabolism and transport to the brain pathway. Copper in food is first reduced by STEAP, which promotes the absorption of copper by intestinal epithelial cells through the action of CTR1. In addition, DMT1 can also transport and absorb copper in certain situations. Subsequently, copper is transported to the portal vein through copper ATP7A, where it binds to plasma proteins such as albumin and copper transporter protein for transport to the liver. A small amount of copper is processed or transported from the liver and released into the bloodstream for circulation into the brain, while excess copper is excreted through bile.

Copper enters the cell through binding with a copper chaperone, enters the mitochondria. Within the mitochondria, COX17 forms a complex with Cu^+^, and subsequently, Cu^+^ could be transferred to SCO1 or COX11, which is involved in the synthesis of cytochrome c oxidase (CcO), the terminal component of the mitochondrial respiratory chain. CcO catalyzes the transfer of electrons from reduced cytochrome c to oxygen, facilitating the oxidative phosphorylation process (McCann C. et al., 2022; Horng et al., 2004; Banci et al., 2008). CCS [Copper Chaperone for superoxide dismutase (SOD)] serves as the copper companion for Superoxide Dismutase 1 (SOD1) and involves transferring copper ions to SOD1, thereby activating the active site of SOD1, which exerts a crucial regulatory role in the cellular redox balance (Boyd et al., 2020; Boulis and Donsante, 2023). In cellular cytoplasm, Metallothionein 1/2 (MT1/2) forms complexes with numerous copper ions, acting as a repository for copper. This interaction is potentially involved in regulating the equilibrium of copper ions within the cell (Tapiero et al., 2003; Ruttkay-Nedecky et al., 2013; Yu et al., 2022). Glutathione (GSH), featuring thiol groups, functions as an antioxidant within cells by directly engaging in enzymatic antioxidant reactions. This involvement manifests anti-oxidative and anti-apoptotic effects. Additionally, GSH may play a role in directly or indirectly regulating the cellular copper pool (Ulrich and Jakob, 2019; Ferguson and Bridge, 2019). Copper transport protein (ATP7A) is expressed in most tissues and organs, while ATP7B is primarily expressed in the liver. ATP7A and ATP7B are located in the trans-Golgi network (TGN), which supply copper to copper-dependent enzymes synthesized in the secretory pathway. The copper efflux rate is determined by the transport or fusion of vesicles containing ATP7A with the plasma membrane (Chun et al., 2017; Janardhanan et al., 2022; Chen et al., 2020) (Figure 1).

## Copper metabolism and internal balance

### Copper metabolism

**FIGURE 2:**
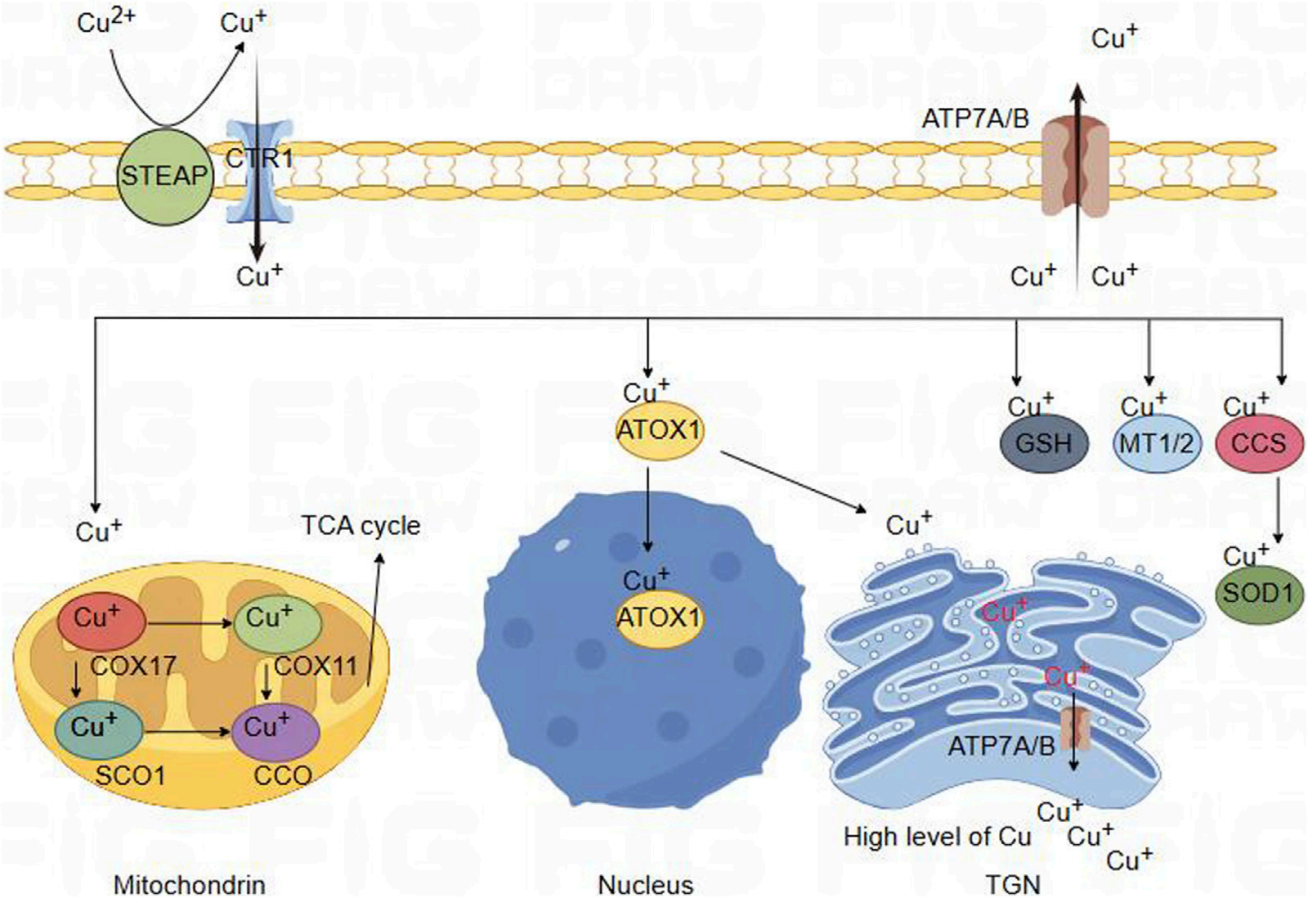
Schematic diagram of copper ion metabolism in body. Copper metabolism in brain cells. Divalent copper cannot be directly absorbed. It is first reduced by STEAP and enters cells through the high affinity of CTR1 for copper absorption on the plasma membrane. Copper binds to copper partners and enters mitochondria, where COX17 forms a complex with copper. Copper can then be transferred to SCO1 or COX11, which participate in the synthesis of CcO, the terminal component of the tricarboxylic acid cycle energy metabolism mitochondrial respiratory chain. Copper could bind with ATOXI into the nucleus and participate in gene expression regulation. It can also be transferred to the Golgi apparatus and released by binding with ATP7A/B, providing copper for copper dependent enzymes synthesized in the secretion pathway. In the cytoplasm, MT1/2 forms complexes with many copper ions, serving as a storage reservoir for copper. This interaction may be involved in regulating the balance of copper ions within cells. GSH can also directly or indirectly participate in regulating cellular copper pools. In addition, CCS acts as a copper partner of SOD1, involving the transfer of copper ions to SOD1. Finally, the efflux of copper is determined by the transport or fusion of ATP7A/B vesicles with the plasma membrane.

Copper is extensively distributed in the biological entities of the natural environment, and it is a crucial trace nutrient for human body. It plays a vital role in numerous redox reactions occurring within human cells, contributing to cellular processes (Chen et al., 2021; Chelyadina et al., 2023). As is well known, Cu is a static cofactor, serving as a catalytic or structural cofactor in the formation of enzymes and proteins, which might be buried and protected within its active sites of enzymes, participating in numerous vital biological activities. Cu is involved in various life processes by constituting enzymes such as cytochrome c oxidase (CCO), SOD, metallothionein (MT), cuproprotein (CP), and lysyl oxidase (LOX). Its participation extends to functions like energy metabolism, antioxidant stress response, vascular formation, and neurotransmitter synthesis (Chen et al., 2020; Tsang et al., 2021; Zhou et al., 2023). Internal copper metabolism is tightly regulated, and extreme deficiency or excess of copper disrupts the copper balance, leading to the onset of diseases and even posing a threat to life (Kirsipuu et al., 2020). Copper deficiency could influence the activity of SOD and the antioxidant capacity of other cellular components, such as iron, selenium, and glutathione (Altarelli et al., 2019; Trist et al., 2021). Copper deficiency may be associated with early pregnancy miscarriage, inflammatory responses, severe anemia, cardiovascular system issues, optic nerve function, and neuro-degenerative changes (Gurnari and Rogers, 2021; Hu et al., 2023; Cendrowska-Pinkosz et al., 2022; Wu et al., 2023). Similarly, copper accumulation could lead to abnormal oxidative metabolism, attacking to the structures and functions of biomolecules such as proteins, which could lead to the occurrence of diseases such as diabetes, neurological disorders, and cardiovascular dysfunctions (Bjørklund et al., 2020; Shi et al., 2023; Gaetke and Chow, 2003). Thus, copper homeostasis is regarded as crucial mechanism for maintaining the balance in the body. The absorption of dietary copper depends on the chemical form of copper and the interaction with dietary components. Daily intake below 0.8 mg/day results in a net loss of copper, while intake above 2.4 mg/day leads to a net increase (Bost et al., 2016). Studies indicates that excessive dietary copper intakes are associated with atherosclerosis, cardiovascular diseases, abdominal aortic calcification, neuronal and mixed neuronal-glial tumors, diabetes, and various neurodegenerative diseases (Yang et al., 2024; Li X. et al., 2023; Liu and Liang, 2024; Eljazzar et al., 2023; Zhang W. et al., 2023; Zhang Y. et al., 2023). In the mammalian digestive tract, copper in food is reduced from its divalent state by the metal-reducing enzyme (STEAP) to a monovalent state. Subsequently, it is absorbed by intestinal cells through the high-affinity copper transporter 1 (CTR1) (Li, 2020). Divalent metal transporter 1 (DMT1) could also transport copper under certain circumstances. The intestinal DMT1 is crucial for the absorption of iron but not essential for the absorption of copper or manganese in mice. A high-iron diet inhibited the intestinal transport of copper through DMT1 (Lin et al., 2015; Sánchez-González et al., 2022; Shawki et al., 2015). As previously reported, copper absorbed in the intestine is transported to the portal vein through ATP7A. In the portal vein, copper binds to plasma proteins such as albumin and transcuprein, facilitating transport to liver, which serves as the central control system for copper homeostasis (Alessi et al., 2021; Horn and Wittung-Stafshede, 2021). Most excess copper is excreted through the bile into the feces, with only a small amount excreted through the kidneys. Impairment of biliary copper excretion could lead to hepatic copper retention (Lenartowicz et al., 2015; Schilsky et al., 2023) (Figure 2).

### Cu protein in mammal metabolism

#### Ferredoxin (Fdx)

In mitochondrial matrix of eukaryotic cells, there is an unstable copper pool, which serves as the location for the assembly of mitochondrial iron-sulfur (Fe/S) clusters (Du et al., 2023). Ferredoxin (FDX) is a vital protein mediator in biological electron transfer reactions, which characterized by the presence of [2Fe-2S] or [4Fe-4S] clusters, allowing it to efficiently accept and release electrons, contributing to its active involvement in oxidation-reduction reactions (Nzuza et al., 2021). Disruption of copper homeostasis may negatively impact the formation of mitochondrial Fe/S clusters, which could lead to impaired iron-sulfur cluster biogenesis, affecting mitochondrial function. The impact extends beyond the respiratory chain, influencing various mitochondrial functions like citric acid cycle, heme biosynthesis, and sulfur-containing amino acid synthesis may be compromised (Ruiz et al., 2021; Cobine et al., 2021). FDX, as an electron donor, engages in various metabolic processes due to its versatile nature, which include participation in the synthesis of steroids, hemoglobin, vitamin D, and the biogenesis of Fe-S clusters in different organisms. Although Fdx1 and Fdx2 share significant functional similarities, they exhibit specific and distinct roles in different physiological pathways (Dreishpoon et al., 2023a; Braymer et al., 2021). Lipoic acid synthase (LIAS) generates lipoic acid cofactors, crucial components for tricarboxylic acid cycle (TCA) cycle enzymes like pyruvate dehydrogenase (PDH). FDX1 acts as an upstream regulator of LIAS, participating in the regulation of the PDH complex. DLAT is a component of the mitochondrial PDH. Loss of FDX1 resulted in the accumulation of pyruvate and α-ketoglutarate, impacting the TCA cycle (Joshi et al., 2023; Dreishpoon et al., 2023b). FDX1 is a crucial regulator of copper ionophore-induced cell death and serves as an upstream regulator of cellular protein lipidation. FDX1 directly binds to LIAS, facilitating its functional interaction with the lipoic acid carrier protein (glycine cleavage system protein H, GCSH), which direct interaction allows FDX1 to regulate protein lipoylation, bypassing indirect modulation of cellular Fe-S cluster biogenesis.

FDX1 promotes the formation of LIAS-GCSH bonds and activation of lipoic acid, which is crucial for cellular processes (Wang Z. et al., 2023; Ke et al., 2023; Schulz et al., 2023). The reductase encoded by FDX1 undergoes protein thioylation regulation during the reduction of Cu^2+^ to Cu^+^, promoting oligomerization of dihydrothioacyl S-acetyltransferase (DLAT). The regulatory mechanism subsequently induces protein toxicity stress, indicating the significant role of FDX1 in copper ion metabolism and cellular stress response (Joshi et al., 2023; Weiler et al., 2020; Li Y. et al., 2023). FDX1 knockdown results in a decrease in the lipoylation level of DLAT and DLST in thyroid cancer cells, which contributes to the alleviation of cell death induced by copper accumulation (Chen G. et al., 2023). FDX1 could regulate the activity of cytochrome c oxidase and mitochondrial respiration, and has multiple targets *in vivo*. FDX1 knockout led to a specific reduction in cytochrome c oxidase, influencing copper and heme A/a3 levels (Zulkifli et al., 2023).

#### Cytochrome c oxidase (COX)

Mitochondrial Cytochrome c Oxidase is located in the inner mitochondrial membrane and serves as a crucial enzyme in the respiratory chain. As the fourth central enzyme complex, also known as Complex IV, it receives electrons on cytochrome C molecules and transfers them to oxygen, generating water and releasing a substantial amount of energy (Blomberg, 2020; Ramzan et al., 2021; Brischigliaro and Zeviani, 2021). Copper serves as a necessary cofactor for COX, with three copper ions needed to construct the dual-core CuA and mononuclear CuB sites, ensuring the enzyme’s stability and activity (Maghool et al., 2019). The assembly of the CuA center necessitates the presence of copper chaperones SCO1, SCO2, and COA6. COA6 knockout in HEK293T cells resulted in decreased activities of both complex I and IV, accompanied by a reduction in mitochondrial membrane potential (Pacheu-Grau et al., 2020). The cellular COX is a complex comprising 13 subunits, and its functionality involves a highly coordinated process. The regulation of COX expression and function is essential for sustaining cellular respiration and energy production (Watson and McStay, 2020). The involvement of Cytochrome c oxidase subunit 5a (COX5a) is essential for preserving regular mitochondrial function.

## Metabolism of copper ions in brain

Copper has been confirmed abundantly present in the brain, playing a crucial role in brain function and development. The subventricular zone (SVZ) in the lateral ventricles, containing a high amount of copper, is the largest neurogenic region in the adult brain and plays a crucial role in neurogenesis (Liu L. L. et al., 2022). Clinical studies confirmed that the severity of brain atrophy is associated with the functional and neurological impairments in patients with Wilson’s disease (WD). Brain volume serves as an indicator of neuro-degeneration induced by copper (Shang et al., 2023). Disruption of copper homeostasis may be a primary cause of various neurological disorders, particularly when elevated copper ion levels occur during brain I/R injury, leading to neuronal damage (Zhang et al., 2020; Chen X. et al., 2023). The potential mechanisms underlying the toxicity induced by the excessive accumulation of free copper within neural cells remain unclear, which are associated with oxidative stress attacks, cell apoptosis, copper death, and other factors (Chen X. et al., 2023; Goto et al., 2020; Gao et al., 2024). In 2022, Peter Tsvetkov, et al. first proposed a novel form of cell death induced by copper ions, which named “cuproptosis” (Kahlson and Dixon, 2022). Emerging evidence supports the facts that copper death is a novel form of cell death involved in regulating cerebral I/R injury. In a rat model of cerebral I/R injury, elevated copper ions and abnormal expression of copper death-related proteins were observed (Guo Q. et al., 2023). Thus, much progress has been made in understanding the pathophysiological mechanisms of copper death in cerebral I/R, it has attracted more and more attention worldwide. It is accepted that the mechanism of I/R is an important driving force for cerebral I/R injury. In this review, we intended to discuss the recent advances in the copper balance.

Endothelial cells within the blood-brain barrier acquire copper from the blood through CTR1 and transport it to the brain parenchyma via ATP7A. Copper homeostasis in the brain involves a complex interplay between uptake, distribution, utilization, and efflux mechanisms (Washington-Hughes et al., 2023; An et al., 2022). Copper ions traverse the blood-brain barrier to enter the brain parenchyma, initially encountering astrocytes. Astrocytes possess the capability to absorb, store, and release copper (Li B. et al., 2024). Copper is allocated to various brain cells according to the demands of different cell types. The copper concentration within astrocytes is the highest, influencing copper homeostasis in the brain. When astrocytes die due to brain injury or aging, copper may be released from astrocytes, potentially exerting adverse effects on neighboring brain cells (Górska et al., 2023; Bhattacharjee et al., 2020). The copper transporter protein CTR1 on the cytoplasmic membrane of brain nerve cells could efficiently uptake copper with high affinity (Wen et al., 2021).

### Biological functions of copper in brain/and neuronal cells

#### Cu protein in brain metabolism

Copper is crucial for the normal functioning of the brain, participating in the synthesis of neurotransmitters such as dopamine and adrenaline. Copper distribution in the brain is uneven, with the highest concentration in the locus coeruleus region of brainstem neurons (Rihel, 2018; Scheiber et al., 2014). This region serves as a primary source of norepinephrine (NA) in CNS. The neurotransmitter NA plays a role in regulating wakefulness, sensory processing, attention, aversion, adaptive stress responses, as well as higher-order cognitive functions and memory (Ma et al., 2023; Kawahara et al., 2021). The copper content is higher in the substantianigra, and the decreased levels of copper in the substantia nigra of Parkinson’s disease (PD) brain further indicate the significant role of copper in this brain region (Bisaglia and Bubacco, 2020). Copper also plays a crucial role in synaptic transmission. In the synaptic cleft, copper could directly or indirectly modulate the activity of neurotransmitter receptors (NMDA, AMPA, GABAA, P2X receptors), influencing neural excitability. Neurotransmission could influence the transport and transmission of copper within neuronal cells (D’Ambrosi and Rossi, 2015; Wang et al., 2020). Copper in brain, besides participating in specific functions mentioned earlier, is also associated with general metabolism, which includes energy metabolism (cytochrome c oxidase, TCA cycle (FDX1)), antioxidant defense (superoxide dismutase containing zinc and copper, copper blue protein), iron metabolism (copper blue protein), neurotransmitter synthesis (dopamine-β-monooxygenase), and neuropeptide synthesis (peptide glycine-α-amidating enzyme) (Liu L. L. et al., 2022; Scheiber et al., 2014; Wang et al., 2020; Masaldan et al., 2018; Li et al., 2022). Neural cells require enzymes and proteins that depend on copper coordination to perform their normal functions. Additionally, some proteins rely on copper for metabolic regulation. Several important proteins expressed in brain and involved in these processes are described as following:

##### Ceruloplasmin (both soluble and GPI-anchored ceruloplasmin)

Genetic mutations in the CP gene result in aceruloplasminemia, a hereditary condition characterized by progressive degeneration of the retina and basal ganglia neurons (Bonaccorsi di Patti et al., 2021). Patients with WD undergo impaired copper excretion from the liver, leading to copper ion deposition in the brain and the manifestation of neurological symptoms, even after undergoing liver transplantation (Xu et al., 2022). Additionally, in a mouse model of permanent middle cerebral artery occlusion-induced cerebral ischemia, mice lacking CP exhibit an increased area of cerebral infarction, leading to more severe oxidative stress damage to lipids and proteins (Ryan et al., 2019).

##### COX

Overexpressing COX5a in the SAMP8 aging mouse model led to improvements in spatial recognition, memory, and hippocampal synaptic plasticity and also restored hippocampal CA1 dendrites, playing a crucial role in modulating age-related cognitive decline (Xiyang et al., 2020). In hypoxia-ischemia (HI) rat model, a manifestation of severe neurological dysfunction, cerebral infarction, cell apoptosis, and notable neuronal loss is concomitant with reduction in COX5a expression. Conversely, COX5a upregulation facilitated neuronal survival post-oxygen-glucose deprivation (OGD), decreasing apoptosis and notably increasing the length of neuronal dendrites (Jiang et al., 2020). In addition, COX6b1 overexpression alleviated I/R-induced hippocampal neuron damage in rat (Yang et al., 2019).

##### Fdx

In a rat MCAO model, a significant upregulation of FDX1 was observed, while interventions with DEX resulted in reduction in FDX1 expression, which alleviates cerebral infarction area and decreases copper-induced neuronal death. Furthermore, in PC12 cells, post-OGD/R treatment led to increased FDX1 expression, causing DLAT oligomerization and triggering copper-induced neuronal death (Wang D. et al., 2023).

##### SOD

The structure, function, and mutations of copper-zinc SOD are associated with neurodegenerative disorders, particularly amyotrophic lateral sclerosis (ALS) (Altobelli et al., 2020; Sanghai and Tranmer, 2021). The activation of immature SOD1 involves in the copper chaperone (Ccs1), which includes Ccs1 delivering copper and promoting the oxidation of the intramolecular disulfide bonds within SOD1, or activation through the entry of copper ions into the sulfur-containing intermediate at the binding site (Fetherolf et al., 2017). In rats subjected to cerebral I/R injury, overexpression of transgenic SOD1 alleviates oxidative stress injury, which exhibited protective effects on nerve cells (Chan et al., 1998). The key to combating cerebral I/R injury is enhancing the activity of endogenous antioxidant enzymes, with SOD1 capable of clearing excessive ROS production (Park et al., 2021). Therefore, external intervention to enhance SOD1 activity could be utilized in the development of protective agents against ischemic injury.

##### Dopamine β-hydroxylase (DBH)

DBH is a member of the copper-dependent monooxygenase family, located in the endoplasmic reticulum, which encoded by the DBH gene and catalyzed the conversion of dopamine to norepinephrine (Juárez-Cedillo et al., 2023). Functionally similar to DBM, and both may be different names for the same enzyme. Dietary copper deficiency in rats leads to a downregulation of dopamine beta-monooxygenase levels in brain tissues (Prohaska and Brokate, 2001). In patients with Menkes disease, the reduction of copper content led to an increased ratio of DBM deficiency in dopamine/norepinephrine, resulting in abnormal behavioral and psychological manifestations (Guthrie et al., 2020; Ohkubo et al., 2019). DBH is the enzyme responsible for producing norepinephrine (NE), while dopamine (DA) and NE are crucial neurotransmitters necessary for normal brain function, which are associated with various diseases such as hypertension, congestive heart failure, Alzheimer’s disease, PD, and Huntington’s disease (Gonzalez-Lopez and Vrana, 2020; Vendelboe et al., 2016).

##### Peptidylglycine α-amidating monooxygenase (PAM)

Peptide hormones are synthesized, stored, and released in the anterior pituitary. The activation of inactive peptide hormone precursors requires the post-translational processing enzyme PAM (Bonnemaison et al., 2016), which converts peptides with extended glycine to amidated products, involved two single-electron transfer steps catalyzed by copper ions at two specific sites. This process is involved in the biosynthesis of peptide hormones (Bäck et al., 2022; Merkler et al., 2022). PAM, an ascorbate- and copper-dependent membrane enzyme that enters secretory granules along with its soluble substrates. Biological studies elucidated the highly conserved mechanism for amidated peptide production and raised many questions about PAM trafficking and the effects of PAM on cytoskeletal organization and gene expression. With the identification of PAM, it showed that PAM was composed of two soluble catalytic cores, including PHMcc (the catalytic core of PHM) and PALcc (the catalytic core of PAL) and a protease-sensitive cytoplasmic domain. Copper ions bound at two sites separated by an 11 Å aqueous cleft participate in the two single electron transfer steps needed to generate a peptidyl-α-hydroxyglycine product (Grechnikova et al., 2020). As previously reported, PAM^−/−^ mice could only survive until mid-pregnancy (Kumar et al., 2016). PAM (+/−) heterozygous mice exhibited anxiety-like behavior, alterations in temperature regulation, and increased susceptibility to seizures, which could be reversed by dietary copper supplementation, suggesting that physiological functions sensitive to PAM genetic constraints could be reversed by copper supplementation (Bousquet-Moore et al., 2010). In primary pituitary cells subjected to 4 h of hypoxia, hypoxia-inducible factor 1α (HIF-1α) exhibited an upregulation, which restricts the ability of PAM to generate amidated peptides, further impacting the enzyme’s activity (Rao et al., 2021).

## The pathophysiological role of copper in cerebral I/R injury

### Cerebral I/R injury and cuproptosis

Stroke is a severe neurological disease, which remains the second leading cause of death (GBD, 2019 Stroke Collaborators, 2021). Early identification, thrombolytic therapy, and emergency intervention for acute ischemic stroke could significantly reduce the incidence and mortality associated with strokes (Herpich and Rincon, 2020). The recovery reperfusion of acute ischemic stroke has a high degree of time dependence (Reziya et al., 2023). In clinical trials, it is challenging to grasp the time window for vascular reconstruction. When the recovery of cerebral blood flow exceeds the time window, it cannot improve the tissue state of reversible damage, and may even deteriorate irreversible sequelas, such as malignant edema and even bleeding (Magoufis et al., 2021). However, the molecular mechanisms remained not fully elucidated, which are associated with oxidative stress, apoptosis, ferroptosis, calcium overload, and copper death. Increasing evidence has showed that copper death participates in brain I/R injury. In cerebral I/R injury, ischemia or hypoxia causes decreases in mitochondrial ATP production, which restricts copper transportation, and results in the of copper accumulation (Cunnane et al., 2020). As previously reported, copper-targeted delivery alleviates neuronal damage in ischemic stroke (Huuskonen et al., 2017). During cerebral I/R, the intracellular copper ion uptake and excretion reached a homeostatic balance, which could be disrupted by mitochondrial respiratory attack and limitation in ATP production, potentially the accumulation of copper ions within the cell and cellular cuproptosis (Lin et al., 2021). Therefore, inhibiting neuronal cuproptosis could decrease the levels of copper ions inside nerve cells, mitigating neuronal injury during cerebral I/R.

### Copper accumulation and cuproptosis

**FIGURE 3:**
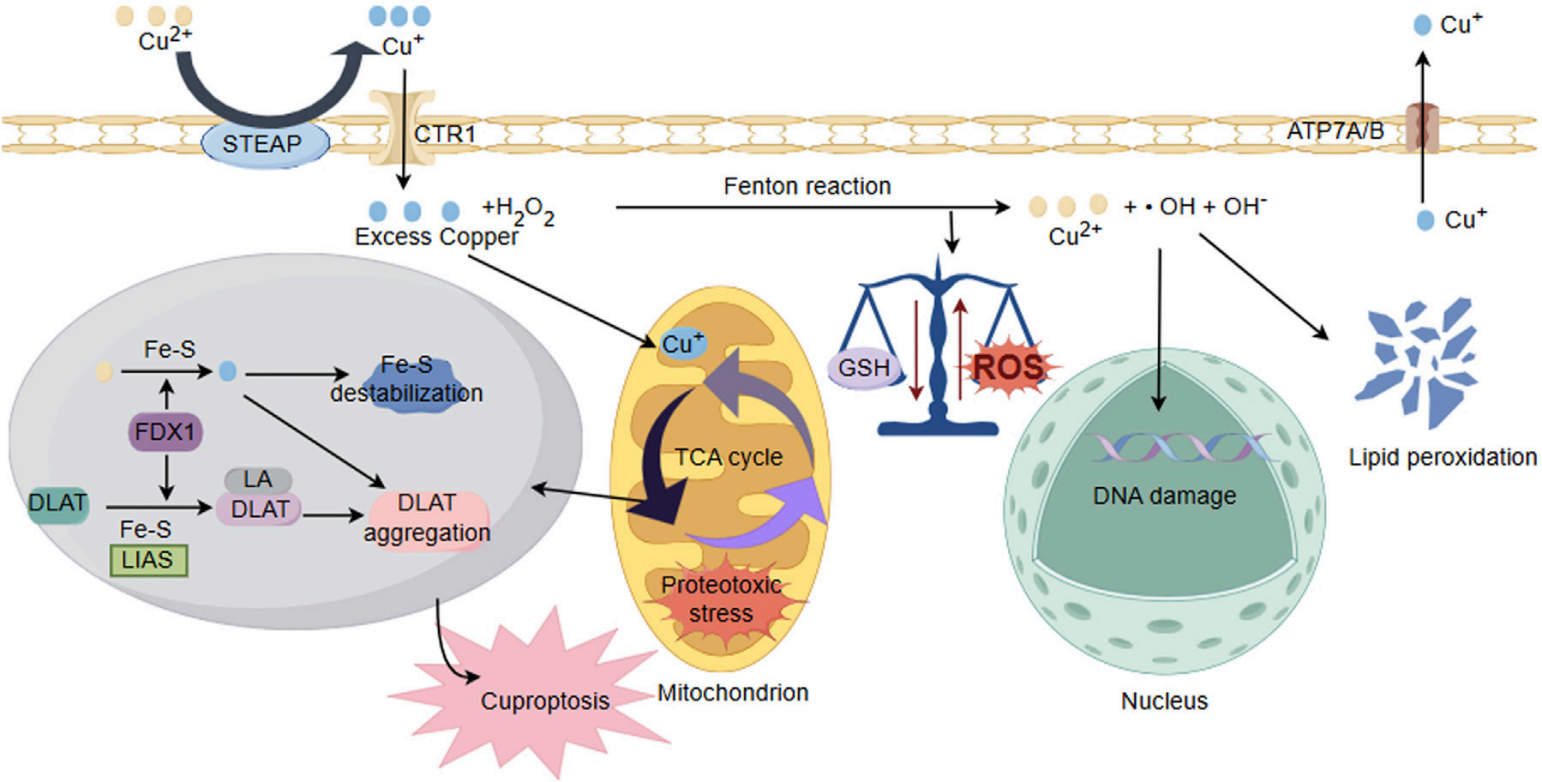
Schematic illustration of copper ion overload inducing neuronal cell injury. Under the action of cytoplasmic oxidoreductases, there is a conversion between Cu^+^ and Cu^2+^, and electron transfer occurs through the Fenton reaction, producing ROS. The accumulation of Cu^2+^ reduces GSH levels and antioxidant capacity, disrupting the balance of cellular redox states and damaging biomolecules including proteins, nucleic acids, and lipids. In addition, ROS could interfere with the synthesis of iron sulfur clusters. Cu binds to thiolated mitochondrial enzymes in the TCA cycle (such as DLAT), inducing the aggregation of these proteins. FDX1/LIAS is an upstream regulator of protein sulfhydrylation. FDX1 reduces Cu^2+^ to more toxic Cu^+^, leading to inhibition of Fe-S cluster protein synthesis, aggregation of mitochondrial proteins and loss of Fe-S clusters, blocking the TCA cycle of the tricarboxylic acid cycle. Collectively, these abnormal processes lead to protein toxicity stress and induce cell death.

Reperfusion of blood supply in ischemic region of the brain after an ischemic stroke yields to irreversible cellular and biochemical consequences, including generation of ROS, expressions of inflammatory cytokines, and inflammation (Lim et al., 2021). Brain tissue is rich in polyunsaturated fatty acids, which is particularly susceptible to attack by ROS and undergoing lipid peroxidation. Intervention with polyunsaturated fatty acids also held potentials for improving neurological outcomes in neonatal hypoxic/ischemic encephalopathy (sHIE) (Martinat et al., 2021; Ng et al., 2022; Dyall et al., 2023). Cu^2+^ exposure reduced levels of glutathione (GSH) and antioxidant capacity while promoting lipid peroxidation in tissues. Some studies indicate that copper (Cu-HCF) nanocomposites catalyze the conversion of reduced glutathione (GSH) to oxidized glutathione within tumor cells, leading to the depletion of GSH (Li C. et al., 2024). The generation of mitochondrial ATP occurs through the process of oxidative phosphorylation (OXPHOS), where the OXPHOS system operates the following five enzyme complexes in the electron transport chain (ETC): Complex I (NADH: ubiquinone oxidoreductase), Complex II (succinate dehydrogenase, SDH), Dimeric Complex III2 (cytochrome bc1 oxidoreductase), and Complex IV (cytochrome c oxidase) (Vercellino and Sazanov, 2022). Due to the presence of two crucial copper sites essential for its functionality, respiratory Complex IV in mitochondria requires copper to sustain the functionality of cytochrome c oxidase (CcO). Therefore, its activity could be easily manipulated by either copper removal/chelation or copper overload, thus influencing the structural and functional aspects of the Electron Transport Chain (ETC) (Ruiz et al., 2021; Swaminathan and Gohil, 2022). Some studies indicate that prolonged exposure to copper in liver and muscles of the viviparous killifish leads to a reduction in energy production, which attribute to copper inhibiting anaerobic pathways and the mitochondrial respiratory chain (Abou Anni et al., 2019). Furthermore, in the liver of patients with Wilson’s disease (WD), copper overload could lead to mitochondrial injury, impairing energy metabolism (Mazi et al., 2020). In cerebral I/R injury, the level of copper ions increases. Cu^+^ directly binds to the sulfur-containing components in the mitochondrial TCA, leading to copper-induced reduction of Fe-S (iron-sulfur) proteins, which triggers protein toxicity stress, inducing copper-dependent cell death. Recent study indicates that Disulfiram (DSF), a widely used drug for controlling alcoholism, possesses anticancer activity by inducing apoptosis in a copper Cu-dependent manner (You et al., 2019). DSF/Cu could accelerate copper death in liver cancer cells (HCC), accompanied by GSH depletion and increased lipid peroxides (Zhang et al., 2024). Taken together, these findings suggest that sustained copper accumulation may induce oxidative stress damage, mitochondrial dysfunction, and the occurrence of cuproptosis in cerebral I/R injury (Figure 3).

### Regulating the level of copper ion and inhibit cuproptosis in cerebral I/R

Clinical studies have found a significant increase in free copper ions in blood samples collected from patients with acute myocardial infarction and successful percutaneous coronary intervention (Hao et al., 2022). Similarly, there is an increase in copper ion levels in the kidney and small intestine models of mice with I/R injury (Akçil et al., 2000; Lee et al., 2019). In a rat cerebral I/R model, the copper ion content in the hippocampal CA1 region of the brain tissue significantly increased compared with the sham group (Shang et al., 2023). In another MCAO rat study, pretreatment with the copper chelator D-penicillamine significantly reduced cerebral infarct volume and copper ion levels which could be improved mitochondrial respiration and membrane potential, possibly exerting its effects by blocking the cuproptosis pathway mediated by FDX1 (Guo Qingduo et al., 2023). Therefore, reducing the increase in copper ion levels in tissues and organs can inhibit the occurrence of cuproptosis. By regulating the copper ion transport, storage, and output processes in neural cells, the copper ion levels in neuronal cells could be reduced, thereby inhibiting the occurrence of cuproptosis. Seeking targeted therapeutic strategies to alleviate I/R injury by specifically reducing cuproptosis is becoming urgent.

### Regulating copper ion levels by blocking the entry of copper ions into CTR1

CTR, located at the plasma membrane and composed of two types of subunits including CTR1 and CTR2, especially which CTR1 belongs to the superfamily of membrane spanning transport proteins responsible for dietary copper homeostasis in mammalian cells (Schoeberl et al., 2022a). Under a normal physiological copper homeostasis, CTR1 is described to be mainly located at the plasma membrane. However, when the cells are medicated with a high amount of Cu, CTR1 is internalized to Early Endosomal Antigen (EEA1)- and Rab5-marked compartments by endocytosis. After removal of extracellular Cu, the transport protein recycles back to the plasma membrane via the slower recycling endosomes (Garmann et al., 2008; Mandal et al., 2020). The missense mutation of the CTR1 coding gene leads to copper deficiency in the central nervous system, thereby affecting the mitochondrial function of nerve cells (Batzios et al., 2022). The increase in copper content during the aging process of the human brain may be attributed to the dysfunction of the brain barrier CTR1, which may be a trigger for neurodegenerative diseases (Haywood and Vaillant, 2014). The increase of intracellular copper is parallel to the expression of CTR1 mRNA. Chakraborty K, et al. found that the differentiation of PC-12 cells (neurons) involves an increase in intracellular copper (Chakraborty et al., 2022). Copper ions must be transported into cells through the transporter protein CTR1, so it is possible to reduce intracellular copper ions by regulating CTR1.

#### Interference in copper and platinum uptake mediated by CTR1

CTR1 is responsible for copper transport through the N-terminal domain of its amino acid residues. Interestingly, CTR1 also affects platinum uptake in tumor cells (Wang X. et al., 2023). Cisplatin, as a competitor to hCtr1 mediated copper transport, leads to a decrease in cellular copper levels (Akerfeldt et al., 2017). Liang et al.'s study reported that cisplatin reduced copper uptake by at least 50% in ovarian cancer cells (Liang et al., 2014). In addition, Pt (II) drugs can also specifically affect Cu (I) homeostasis by interfering with the rapid exchange of Cu (I) between Atox1 and Cu ATPases (Lasorsa et al., 2019). Which indicated that interference existed between copper and platinum that are substrates for cellular uptake mediated by CTR1. Several *in vitro* studies have disclosed that platinum drugs disrupt cellular copper uptake. In the ovarian cancer cell lines A2780 and the cisplatin-resistant A2780cis cells, utilizing cisplatin treatment exhibited a significant decrease in copper content compared with untreated cells (Schoeberl et al., 2022b). Therefore, platinum based drugs may become a backup drug for intervening in elevated copper ion levels in the future, by reducing intracellular copper ion concentration and inhibiting copper induced cell death.

#### Factors influencing CTR1 expression

In the study of improving the anti-tumor efficacy of platinum-based drugs, upregulation of CTR1 expression can be achieved. NSCLC cells, through glucose restriction, upregulate ROS and induce CTR1 expression in AMPK. Similarly, mice fed a low carbohydrate ketogenic diet (glucose restriction) showed an increase in CTR1 expression in tumor tissue, significantly enhancing the efficacy of cisplatin (Zhang et al., 2022). Changes in the expression of CTR1 within cells can directly affect the transport of copper ions. Downregulation of CTR1 expression reduces copper ion uptake. In the investigation of anti-pancreatic cancer, Song, et al. used targeted CTR1 mRNA therapy to effectively inhibit the expression of CTR1 in PANC-1 cells, reduce copper intake, and delay the progress of pancreatic cancer (Song et al., 2021). In a rat brain I/R injury model, the expression of intracellular CTR1 is downregulated, the concentration of copper ions decreases, and copper induced neuronal death is alleviated, effectively protecting neural function (Strenkert et al., 2024).

However, there are many factors that affect the expression of CTR1, such as the regulation of gene transcription encoded by CTR1. Treatment of M21 melanoma cell lines with the Sp1 selective inhibitor Plicamycin for 24 h or knockout of the Sp1 gene resulted in a decrease in the expression of Sp1 and CTR1, as well as a decrease in intracellular copper ion levels and an increase in cell survival rate (Lin et al., 2014). In U2OS cells, SP1 can directly bind to the CTR1 promoter. Overexpression SP1 stimulates CTR1 expression, increases copper ions and inhibits nuclear translocation of (SP1), knockout of p53 promotes CTR1 expression and cisplatin uptake, while p53 overexpression inhibits CTR1 expression and cisplatin uptake (Yong et al., 2023). In VPS35 knockout in HeLa cells, the results showed a significant decrease in the abundance of CTR1 on the cell surface and a significant decrease in copper ion levels in the absence of reverse transcriptase function, indicating that reverse transcriptase affects the transport of copper by CTR1 (Curnock and Cullen, 2020). Carine White’s et al. found that RAW264.7 macrophages pre-exposed to hypoxic conditions showed a significant increase in copper ion uptake compared to normoxia (White et al., 2009). Experimental evidence points that the copper uptake of Caco-2 cells under hypoxia is five times higher than normoxia, and the increase in copper uptake in Caco-2 cells under hypoxic conditions is related to the increase in Ctr1mRNA expression (Pourvali et al., 2012). In addition, studies have found that the expression of CTR1 gene is regulated by hypoxia inducible factor (HIF) and Myc transcription (Feng et al., 2009; Porcu et al., 2018).

After the transcription of the CTR1 gene, the stability of its protein can be translated in order to perform its function. Jianping Guo, et al. found that Nedd4l can induce CTR1 ubiquitination and subsequently degrade CTR1 (Guo et al., 2021). Nedd4l can negatively regulate CTR1 copper signaling to regulate AKT kinase activity and reduce tumor occurrence. Based on CRISPR/Cas9, the upstream kinase of CTR1 was identified through transcriptome screening. It was found that AMPK can enhance the localization of CTR1 membrane, and phosphorylation and stabilization of CTR1 are present and play a role in the plasma membrane (Zhang X. et al., 2023). CTR1 is essential for the activation of the MAPK signaling pathway by ligands of three major receptor tyrosine kinases (RTKs): FGF, PDGF, and EGF (Tsai et al., 2012). In summary, during cerebral I/R, modulation of post-translational modifications of CTR1, such as ubiquitination and phosphorylation, could influence its function and inhibit neuronal uptake of copper ions.

#### Factors influencing CTR1 activity

X-ray diffraction shows that CTR1 adopts a homologous trimeric structure similar to Cu^+^ selective ion channels. Two layers of cysteine triad form a selective filter that coordinates two bound copper ions near the extracellular entrance (Ren et al., 2019). Changes in the activity of CTR1 in cells may interfere with intracellular copper ion levels. Initially, it was believed that, similar to other ion channels, its activity could be altered by affecting energy metabolism. However, studies have shown that both mammalian and yeast CTR families lack clear ATP binding domains, and CTR1 mediated copper uptake may not be energy dependent (Margaret et al., 2023). Moreover, Peter Tsvetkov et al.'s research show that mitochondrial respiration is involved in copper-induced cell death, on the contrary, disrupting ATP generation with mitochondrial uncoupling agents showed no effect on copper-induced cell death (Tsvetkov et al., 2022). However, changes in the extracellular environment may affect CTR1 activity. The N-terminal region of the human copper transporter protein Ctr1 exhibits pH and metal oxidation state-dependent multi-metal binding capabilities (Lee et al., 2002). Experimental findings on the impact of extracellular pH, Na, and K+ on copper absorption indicated a significant increase in copper accumulation in Hek5 cells at pH 5.5 and 5.7 compared to pH 7.5. Compared with low K buffer, incubation of Hek5 cells in high K buffer significantly increased copper accumulation (Ren et al., 2019). Collectively, these findings suggest that acidic or high potassium could activate CTR1 activity, once neuronal cells undergo anaerobic glycolysis, acidosis, and high potassium levels, changes in the cellular microenvironment might cause stimulation on CTR1 activity, which led to increased copper ion uptake and subsequent copper-induced neuronal cell death. Furthermore, emerging studies indicate that the endogenous retrovirus envelope glycoprotein Refrex1 could interact with CTR1, reducing its transport activity to regulate copper uptake (Nardella et al., 2022). These results indicated that altering CTR1 activity through changes in the extracellular environment and protein interactions represents a potential target against copper-induced cell death due to elevated copper ion levels.

### Regulating copper ion levels by modulating the copper efflux pathway ATP7A/B

ATP7A/B is a Cu + transporter ATPase, which maintains copper homeostasis within cells and involves the transmembrane transfer of Cu + ions from the cytoplasm to receptor proteins, and requires ATP hydrolysis (Tury et al., 2023). Mutations in ATP7A and ATP7B (more specific to liver where it plays an important role to deliver Cu to apoceruloplasmin) are the basis for MD and WD, respectively. ATP7A facilitates the extrusion of copper through the cell membrane to maintain cellular copper homeostasis. The copper accumulation in motor neurons is the pathogenic mechanism associated with ATP7A in the hereditary motor neuropathy dHMN (Tadini-Buoninsegni and Smeazzetto, 2017). However, some results indicated that genetic mutations in the genes encoding copper efflux proteins ATP7A/B could lead to functional impairment, which might impair Cu^+^ efflux within cells, copper overload, and induction of copper related cell death. A study in a Wilson Disease (WD) mouse model demonstrated that a decrease in Fe-S proteins, protein sulfhydration, and expression of copper death-related proteins showed shared occurrence of copper-induced cell death mechanisms in genetic models (Dong et al., 2021). Hepatolenticular degeneration is a mutation in the ATP7B gene, where copper ions continue to accumulate in brain, liver, and other tissues. The intervention strategy for WD primarily depended on the utilization of copper ion chelators such as D-penicillamine and tetrathiomolybdate (TTM) (Litwin et al., 2019). Studies have revealed that heterogeneous nuclear ribonucleoprotein hnRNPA2/B1 regulates Cu + homeostasis by modulating the Cu(I)-transporting protein ATP7A (McCann Courtney J. et al., 2022). In Hela cells, hnRNPA2/B1 downregulation increases ATP7A mRNA and protein levels, significantly decreasing cellular copper levels (Natera-de Benito et al., 2021). In HPrEC cells, certain anticancer drugs, like flavonoids, disrupt the expression of the copper transport gene ATP7A, leading to impaired copper ion transport and subsequent cell death (McCann C. J. et al., 2022). Furthermore, in human neuroblastoma SH-SY5Y cells, NaHS significantly reduces the levels of ATP7A, promotes intracellular Cu + accumulation, which induce cellular toxicity (Goto et al., 2020). COMMD1 performs intracellular ATP7A/B stability chaperone function, and copper levels increase in cells lacking functional COMMD1 protein (Singla et al., 2021). Therefore, by regulating the expression of ATP7A/B, it is possible to influence the intracellular copper ion levels. In the study of cerebral I/R in rats, it was found that the expression of ATP7B was significantly downregulated compared to the control group, and the intracellular copper ion level was increased. Dexmedetomidine pretreatment in cerebral I/R causes increase in ATP7B strongly expressed in brain, reduce intracellular copper ions, and inhibit neuronal copper death (Guo Qingduo et al., 2023). These results indicate that overexpression of ATP7A/B in neuronal cells, increased excretion of copper ions, and inhibition of copper induced neuronal cell death, which provided significant implications for the treatment of cerebral I/R injury.

## Future prospectives

Taken together, the primary treatment approach for stroke is early perfusion to alleviate ischemic damage. However, early reperfusion could result in I/R injury, necessitating urgent exploration of therapeutic strategies to mitigate these assaults. During cerebral I/R, there is an elevation in copper ion levels, abnormal expression of copper death-related proteins, and the occurrence of copper-induced cell death in neurons. Understanding the physiological metabolism of copper ions in the brain, reducing cerebral copper ion levels during I/R injury aims to alleviate copper-induced cell death, offering a novel avenue for treating cerebral ischemic injury. While current research on cuproptosis primarily focuses on tumor treatment and drug resistance mechanisms, lessons from various strategies to decrease intracellular copper ions in cancer research could be applied to inhibit cuproptosis, contributing to studies aimed at alleviating cerebral I/R injury. Recently, although substantial progress has been made in unraveling the mechanisms of cuproptosis during cerebral I/R, there are still no effective remedies available for reliving cerebral I/R injury. This requires further studies and novel approaches to facilitate the molecular features and excavate more rational strategies.
